# Effects of a New Group of Antidiabetic Drugs in Metabolic Diseases

**DOI:** 10.31083/j.rcm2402036

**Published:** 2023-02-02

**Authors:** Jaime Sanz-Cánovas, Michele Ricci, Lidia Cobos-Palacios, Almudena López-Sampalo, Halbert Hernández-Negrín, María Vázquez-Márquez, Juan José Mancebo-Sevilla, Elena Álvarez-Recio, María Dolores López-Carmona, Miguel Ángel Pérez-Velasco, Luis Miguel Pérez-Belmonte, Ricardo Gómez-Huelgas, Maria-Rosa Bernal-López

**Affiliations:** ^1^Unidad de Gestión Clínica de Medicina Interna, Hospital Regional Universitario de Málaga, Universidad de Málaga (UMA), 29010 Málaga, Spain; ^2^Instituto de Investigación Biomédica de Málaga (IBIMA-Plataforma Bionand), 29590 Málaga, Spain; ^3^Centro de Investigación Biomédica en Red Fisiopatología de la Obesidad y Nutrición (CIBERobn), Instituto de Salud Carlos III, 28029 Madrid, Spain

**Keywords:** SGLT2 inhibitors, metabolic diseases, type 2 diabetes mellitus

## Abstract

The prevalence of type 2 diabetes mellitus (T2DM) is rising in the general population. This increase leads to higher cardiovascular risk, with cardiovascular diseases being the main cause of death in diabetic patients. New therapeutic weapons for diabetes mellitus are now available. Sodium-glucose cotransporter type 2 (SGLT2) inhibitors are novel drugs that are widely used due to their strong benefit in preventing hospitalization for decompensated heart failure and renal protection, limiting the deterioration of the glomerular filtration rate, independently of the presence of diabetes mellitus. These drugs have also shown benefit in the prevention of atherosclerotic cardiovascular events and cardiovascular mortality in diabetic patients with established cardiovascular disease. On the other hand, patients with T2DM usually present a high burden of associated comorbidities. Some of these entities are arterial hypertension, dyslipidemia, hyperuricemia, obesity, non-alcoholic fatty liver disease (NAFLD), polycystic ovary syndrome (PCOS), vascular aging, respiratory diseases, or osteoporosis and fractures. Healthcare professionals should treat these patients from an integral point of view, and not manage each pathology separately. Therefore, as potential mechanisms of SGLT2 inhibitors in metabolic diseases have not been fully reviewed, we conducted this review to know the current evidence of the use and effect of SGLT2 inhibitors on these metabolic diseases.

## 1. Introduction

Type 2 diabetes mellitus (T2DM) is a chronic disease whose prevalence has been steadily increasing over recent decades. The latest-published data estimate that approximately 463 million adults suffer from T2DM worldwide, three more times than two decades ago [1], and these figures are set to increase at a very high rate, estimated to reach 700 million by 2045 [2]. Currently, it is being observed that countries experiencing prompt epidemiological transitions are presenting more rapid increases in this disease. This may be due to recent changes in the lifestyles of this population, mainly a sedentary lifestyle and worse nutritional quality [3]. Diabetes mellitus and obesity are two closely related comorbidities, in which lifestyles have an influence on their development. They both generate a great impact on health and the economy [4]. Obesity is defined as an increase in body mass index (BMI) greater than or equal to 30 kg/$m^{2}$, and it can be divided into different degrees obesity implies an increased risk for developing T2DM and cardiovascular events with respect to the population with a normal BMI [5, 6].

The main dietary styles observed in the development of T2DM and obesity are the increased consumption of hypercaloric foods of low nutritional quality, characterized by refined cereals, animal fats and proteins, added sugars, sodium, and trans fats. At the same time, the consumption of legumes, whole grains, vegetables, and fruits has decreased considerably, with a simultaneous decrease in physical activity [7, 8]. Other risk factors for developing T2DM are not modifiable, such as age and genetics. Therefore, our main efforts should be focused on promoting healthy diets and lifestyle to prevent the onset of T2DM [3]. In this population, foods rich in fruits, vegetables, whole grains, nuts, or dairy are recommended, preferably under the supervision of doctors and nutritionists [8, 9, 10]. The Mediterranean diet is a good example of a favorable diet both for diabetic patients and for preventing the development of T2DM, with studies recommending good adherence to the Mediterranean diet in this patient profile [11, 12].

Over recent decades, a wide arsenal has been developed to treat T2DM. More and more specific treatments are becoming available, which depend not only on glycemic control, but also on the characteristics of the patient as a whole. Patient comorbidities, risk of hypoglycemia, effects on body weight, side effects, costs, and patient preferences become important when choosing the best antidiabetic treatment for a patient [13].

Sodium-glucose cotransporter type 2 (SGLT2) inhibitors are a new group of oral drugs with multiple important benefits that are now widely used. Seven isoforms of the sodium-glucose cotransporter have been described to date, although their expressions and functions in some of them are elusive [14]. At the renal level, two isoforms, Sodium-glucose cotransporter type 2 (SGLT2) and Sodium-glucose cotransporter type 1 (SGLT1), were found. SGLT2 is the main isoform expressed in the proximal convoluted tubules, and it is responsible for the uptake of approximately 90% of filtered glucose at the renal level. It is also found in the alpha cells of the human pancreas regulating glucagon release [15]. It has a high capacity, but low affinity, for glucose. On the other hand, SGLT1 has a high affinity, but low capacity, for glucose. SGLT1 is expressed mainly in the gastrointestinal tract and liver and, to a lesser extent, in the kidney [16].

SGLT2 may be elevated in patients with T2DM [17]. SGLT2 inhibitors act at the level of the proximal convoluted tubule, blocking this cotransporter and favoring the renal elimination of glucose and sodium, and secondary to the mechanism described, these drugs have a diuretic effect [18]. The glucose-lowering effect of these drugs is independent of pancreatic beta-cell function and insulin sensitivity. This fact is ideal in situations of T2DM progression and the patient’s metabolic milieu [19, 20, 21].

Canagliflozin, dapagliflozin, empagliflozin, and ertugliflozin are the SLGT-2 inhibitors currently available. Although they are drugs with the same mechanism of action, there are some differences between them in the various clinical trials published. Recently, several clinical trials with SGLT2 inhibitors have shown that they reduce heart failure (HF) hospitalization as well as cardiovascular death and major adverse cardiovascular events in patients with T2DM at high cardiovascular risk [22, 23, 24, 25, 26, 27] (Table 1). The beneficial effect of SGLT2 inhibitors in HF has subsequently been corroborated in patients with HF and reduced or preserved LVEF, irrespective of the presence of diabetes [28, 29, 30, 31] (Table 2). In addition, SGLT2 inhibitors have shown promising results in patients with a broad spectrum of chronic kidney disease, with a significant reduction in HF hospitalizations, even in populations without diabetes [27, 31] (Table 3).

**Table 1.** **Cardiovascular safety studies with SGLT2 inhibitors in patients with diabetes**.

Clinical trial	EMPA-REG	CANVAS-R	CREDENCE	DECLARE-TIMI	VERTIS CV
Drug	Empaglifozin	Canaglifozin	Canaglifozin	Dapaglifozin	Ertuglifozin
Heart failure hospitalization	0.65	0.67	0.61	0.73	0.70
	0.50–0.85	0.52–0.87	0.47–0.80	0.61–0.88	0.54–0.90
Cardiovascular death or heart failure hospitalization	0.66	0.78	0.69	0.83	0.88
	0.55–0.79	0.67–0.91	0.57–0.83	0.73–0.95	0.75–1.03
Major adverse cardiovascular events	0.86	0.86	0.80	0.93	0.97
	0.74–0.99	0.75–0.97	0.67–0.95	0.84–1.03	0.85–1.11

EMPA-REG, Empaglifozin Cardiovascular Outcomes, and Mortality in Type 2 Diaebtes; CANVAS-R, Canaglifozin Cardiovascular Assessment Study-Renal; CREDENCE, Canaglifozin and Renal Outcomes in Type 2 Diabetes and Nephropathy; DECLARE-TIMI, Dapagliflozin Effect on CardiovascuLAR Events-Thrombolysis in Myocardial Infarction; VERTIS CV, Evaluation of Ertugliflozin Efficacy and Safety Cardiovascular Outcomes Trial.

**Table 2.** **Cardiovascular outcomes with SGLT2 inhibitors in patients with heart failure**.

Clinical trial	DAPA-HF	EMPEROR-Reduced	EMPEROR-Preserved	DAPA-CKD
Drug	Dapaglifozin	Empaglifozin	Empaglifozin	Dapaglifozin
Heart failure hospitalization	0.70	0.69	0.71	-
	0.59–083	0.59–0.81	0.60–0.83	
Heart failure hospitalization or cardiovascular death	0.74	0.75	0.79	0.71
	0.65–0.85	0.65–0.86	0.69–0.90	0.55–0.92
Cardiovascular death	0.82	0.92	0.91	0.81
	0.69–0.98	0.75–1.12	0.76–1.09	0.58–1.12
All-cause mortality	0.83	0.92	1.00	0.69
	0.71–0.97	0.77–1.10	0.87–1.15	0.53–0.88

DAPA-HF, Dapagliflozin and Prevention of Adverse Outcomes in Heart Failure; EMPEROR-Reduced, Empagliflozin Outcome Trial in Patients with Chronic Heart Failure with Reduced Ejection Fraction; EMPEROR-Preserved, Empagliflozin Outcome Trial in Patients with Chronic Heart Failure with Preserved Ejection Fraction; DAPA-CKD, Dapagliflozin and Prevention of Adverse Outcomes in Chronic Kidney Disease.

**Table 3.** **Renal outcomes with SGLT2 inhibitors in patients with chronic kidney disease**.

	CREDENCE	DAPA-CKD
Drug	Canaglifozin	Dapaglifozin
Renal events*	0.66	0.56
	(0.53–0.81)	(0.45–0.68)
Other primary outcomes**	0.70	0.61
	(0.59–0.82)	(0.51–0.72)

*CREDENCE: End-stage renal disease (dialysis, renal transplant, or estimated glomerular filtration rate (eGFR) <15 mL/min/$m^{2}$). *DAPA-CKD: glomerular filtration rate (GFR) reduction ≥50%, end-stage renal disease, or renal death. **CREDENCE and DAPA-CKD: GFR reduction ≥50%, end-stage renal disease, or death from renal or cardiovascular causes.

Once T2DM is established, diabetic patients are more prone to develop cardiometabolic comorbidities, which increase cardiovascular risk. Therefore, diabetic patients should be thoroughly evaluated to control cardiovascular risks [32]. Due to the high burden of comorbidities in patients with heart failure and diabetes mellitus, and the widespread use of SGLT2 inhibitors in these patients, we now review how SGLT2 inhibitors act on other comorbidities.

## 2. Obesity

There are studies that have evaluated the associations between BMI trajectories and T2DM incidence [33, 34, 35, 36]. SGLT2 inhibitors have a beneficial effect on weight reduction in overweight and obese patients with T2DM. Weight reduction occurs at the expense of body fat reduction [37]. This effect has been studied in patients during two years of follow-up, therefore, there is not enough evidence to know if it can be sustained for years [38]. There are different mechanisms by which SGLT2 inhibitors reduce body weight. Firstly, weight loss may be due to calorie loss related to increased urinary glucose excretion. Secondly, glucosuria induces an increase in glucagon concentrations, which may influence lipolysis and ketone body levels. This mobilization of accumulated fat would influence body weight reduction. Finally, the leptin/adiponectin ratio, used as a marker of insulin resistance in patients without T2DM, decreases during treatment with these drugs [37].

Several clinical trials have studied the effect of weight reduction in overweight and obese patients without T2DM. SGLT2 inhibitors could be used in these patients, provided that they have a low risk of urinary and genital infections, mostly on the reduction of body mass index. Some meta-analysis showed that SGLT2 monotherapy was associated with significant reduction in body weight of –2.32 kg, compared to –1.01 kg for placebo [39]. The SGLT2 inhibitors group had statistically significant reductions in absolute changes in body weight (mean differences (MD): –1.42 kg, 95% confidence interval (CI): –1.70 to –1.14; *p *< 0.00001) and BMI (MD: –0.47 kg/$m^{2}$, 95% CI: –0.63 to –0.31; *p *< 0.00001) compared to placebo in this meta-analysis [40]. A reduction in waist circumference has also been observed in overweight/obese, non-diabetic people treated with SGLT2 inhibitors (weighted mean difference (WMD) = –1.94, *p* = 0.03) [41]. More studies are needed to strengthen these data.

## 3. Hyperlipidemia

T2DM and hyperlipidemia are two commonly coexisting entities. Approximately 60% of patients with T2DM have hypertriglyceridemia. The effect of SGLT inhibitors on lipid metabolism has been studied in several studies. In different systematic reviews and meta-analyses, a significant reduction in triglyceride levels as well as an increase in high-density lipoproteins-Cholesterol (HDL)-cholesterol, total cholesterol, low-density lipoproteins Cholesterol (LDL)-cholesterol and non-HDL-cholesterol has been found in patients treated with SGLT2 inhibitors [42].

In another recent meta-analysis of 36 included studies on the effect of SGLT inhibitors on weight and lipid metabolism in patients with diabetes mellitus, it was observed that SGLT inhibitors and placebo were not associated with significantly different serum cholesterol levels. SGLT inhibitors did reduce serum triglyceride levels but increased serum high- and low-density lipoprotein cholesterol levels [43]. According to experts, in patients with diabetic dyslipidemia, the use of SGLT2 inhibitors produces a small increase in LDL-C and HDL-C levels and a small decrease in triglyceride concentration. This increase in LDL-C concentration is combined with a reduction in small, dense atherogenic LDL particles, an effect that may play a role in reducing cardiovascular risk. Due to this, SGLT2 inhibitors could improve all factors of diabetic dyslipidemia (triglycerides, HDL-C, small and dense LDL particles), participating in a reduction of residual cardiovascular risk [44].

## 4. Arterial Hypertension

Arterial hypertension is usually defined by the presence of a chronic elevation of systemic blood pressure above a certain threshold value. In patients with T2DM this threshold is 130–139/80–85 mmHg. SGLT2 inhibitors are not currently indicated as antihypertensive agents, but their effect lowering systolic and diastolic blood pressure has been observed in normotensive subjects as well as in hypertensive and normotensive diabetic patients. This hypotensive effect may be an important additional clinical advantage for patients with T2DM [45, 46]. Below, we describe the possible mechanisms of action of SGLT2 inhibitors related to blood pressure control.

The effect of SGLT2 inhibitors results in increased osmotic diuresis and mild natriuresis. This leads to a reduction in plasma volume and a decrease in blood pressure [47, 48]. On the other hand, the reduction in body weight due to the use of SGLT2 inhibitors may lead to reductions in blood pressure in the medium to long term [49]. Another mechanism by which SGLT2 inhibitors may reduce blood pressure is local inhibition of the renin-angiotensin-aldosterone system secondary to increased sodium delivery to the juxtaglomerular apparatus [47, 50]. Other mechanisms, still under study, that may support blood pressure control include possible indirect effects on nitric oxide release, secondary to the reduction in oxidative stress caused by improved glycemic control [49, 51].

The beneficial effect of SGLT2 inhibitors on blood pressure is not exclusively limited to patients with T2DM and overweight or obesity. There are already published studies in which empaglifozin 10 mg shows a significant reduction in daytime (coefficient –0.5; adjusted (adj). $R^{2}$: 0.36; *p* = 0.0007) and night-time (coefficient –0.6; adj. $R^{2}$: 0.33; *p* = 0.001) ambulatory blood pressure in normotensive non-diabetic patients, and it also decreased 24-h systolic and diastolic blood pressure significantly by –5 ± 7 mmHg (*p *< 0.001) and –2 ± 6 mmHg (*p* = 0.03) [52]. Finally, the effect of SGLT2 inhibitors is being studied for use in non-diabetic patients with resistant hypertension, defined as blood pressure above target despite being on 3 or more antihypertensive medications at optimal doses [53].

## 5. Hyperuricemia

Hyperuricemia is defined as a disorder of purine metabolism in which serum uric acid levels are increased and can lead to gout attacks. Uric acid is often increased in patients with T2DM compared to healthy population. Hyperuricemia causes significant morbidity and mortality in the form of renal and cardiovascular disease, and its reduction in patients with T2DM may reduce microvascular and macrovascular complications [54, 55].

SGLT2 inhibitors have been shown to decrease uric acid levels in several clinical studies [56]. Through the expression of glucose transporter 9 isoform 2 in the renal tubules, there is an elevation in the excretion of D-glucose and uric acid in the urine. This is the main known mechanism by which SGLT2 inhibitors decrease serum uric acid levels [57, 58].

The decrease in serum urate levels with the use of SGLT2 inhibitors has also been demonstrated in patients without T2DM compared to placebo as a recent meta-analysis showed (–91.38 μmol/L; 95% CI: –126.53 to –56.24), although further randomized controlled trials are now recommended to confirm these data [59]. In addition, a recent meta-analysis showed that SGLT2 inhibitors may not only reduce uric acid levels, but also potentially prevent gout-related adverse events in people with T2DM (hazard ratio (HR) 0.70, 95% CI: 0.59 to 0.84, *p *< 0.001, $I^{2}$ = 84%) [60].

## 6. Non-Alcoholic Fatty Liver Disease (NAFLD)

NAFLD is one of the leading causes of chronic liver disease worldwide. In our environment it has a prevalence of around 30%, and as it is an asymptomatic pathology, it is usually diagnosed in advanced stages. It is a common comorbidity found in patients with T2DM and/or metabolic syndrome [61].

Previous antidiabetic drugs such as pioglitazone have shown significant improvement in liver profile and liver histology in both diabetic and non-diabetic patients with NAFLD. However, new anti-diabetic therapies, such as SGLT2 inhibitors, are being studied to assess their effect in NAFLD. In a recent meta-analysis, SGLT2 inhibitors trended towards reduced steatosis (standard mean difference (SMD) –4.64, *p* = 0.06), nevertheless, these results were non-significant [62]. Another meta-analysis has shown that alanine transaminase (WMD –5.36 [95% CI: –8.86 to –1.85], *p* = 0.003) and aspartate transaminase (weighted mean difference –2.56 [95% CI: –3.83 to –1.29], *p *< 0.0001) levels are significantly reduced in patients with NAFLD and T2DM with the use of SGLT2 inhibitors versus other antidiabetic treatments, including pioglitazone [63]. Furthermore, three randomized control trials (RCTs) have shown how the use of SGLT2 inhibitors can significantly improve the liver function compared to other anti-diabetic drugs, two of them with ipraglifozin [64, 65], and another one with dapaglifozin, where liver enzimes were decreased at 24 weeks in the dapaglifozin group, regardless of glucose lowering or weight loss [66].

Thus, although lifestyle interventions are the main treatment for NAFLD, the use of SGLT2 inhibitors appears to have potential efficacy on biochemical and histological parameters [67]. However, further studies are currently recommended to understand the mechanisms by which SGLT2 inhibitors have these beneficial effects [68].

## 7. Polycystic Ovary Syndrome (PCOS) 

PCOS is the most common endocrinological condition in women of reproductive age. Metabolic and cardiovascular complications are related to this entity, and its association with T2DM is common [69]. The main treatment consists of lifestyle intervention and drugs as metformin and oral contraceptive pills. These measures do not successfully address the long-term metabolic consequences in PCOS patients. Therefore, SGLT2 inhibitors could be a new treatment option due to beneficial glycemic and cardiovascular effects, which are often an issue for patients with PCOS [70].

Studies available on the use of SGLT2 in PCOS are currently scarce. A recent randomized open-label, noninferiority trial showed that canagliflozin was not inferior to metformin in PCOS patients with insulin resistance (least-squares mean difference –0.81% [95% CI: –2.13 to 0.51]) [71]. Another clinical trial showed a significant improvement in anthropometric measures and body composition, overweight, and obesity in women affected by PCOS treated with empaglifozin compared to metformin, differences confirmed in linear regression analysis after adjustment for relevant covariates [72]. Finally, a clinical trial with licoglifozin, a dual sodium-glucose cotransporter 1/2 inhibitor, showed no effect on body weight, but licoglifozin led to a 6% reduction in body weight in obese patients treated for 12 weeks [73].

Although SGLT2 inhibitors are not currently approved for the treatment of PCOS, and more randomized clinical trials are still needed, this family of anti-diabetic drugs could be useful for PCOS patients due to beneficial effects on blood glucose and the cardiovascular system, which are often a problem in women affected by PCOS, improving comorbidities such as hypertension, dyslipidemia, diabetes mellitus, hyperuricemia, overweight, or obesity [70].

## 8. Vascular Aging

Vascular aging consists in the organic and functional modifications produced in blood vessels. Arterial stiffness, atherosclerosis, vascular calcification, and high levels of β-amyloid are involved in the development of vascular aging. Arterial stiffness is a cardiovascular risk factor, frequently found in patients with T2DM, and associated with the occurrence of cardiovascular events. SGLT2 inhibitors have been shown in multiple clinical trials to improve arterial stiffness and vascular resistance by reducing blood pressure [74]. These drugs reduce arterial stiffness and vascular resistance due to the decrease in endothelial cell activation, stimulating direct vasorelaxation and enhancing endothelial dysfunction or expression of proatherogenic molecules and cells [75].

In terms of clinical evidence of the effect of SGLT2 inhibitors on vascular aging, dapaglifozin [76, 77, 78], empaglifozin [79], and canaglifozin [80] have shown beneficial effects in diabetic patients. Empaglifozin improved pulse wave velocity (PWV) and β-stiffness compared to metformin [by 15.8% (*p *< 0.01) and by 36.6% (*p *< 0.05), respectively] [80]. Furthermore, a study showed there was a significant difference in change of estimated 24-h PWV (–0.16 ± 0.32 versus 0.02 ± 0.27; *p* = 0.007) favoring dapagliflozin versus placebo [81]. Finally, a recent meta-analysis showed that SGLT2 inhibitors do not decrease PWV in patients with established cardiovascular disease or cardiovascular risk factors, but cause a slight and significant decrease in PWV in patients with T2DM [82]. It is important to highlight that a significant correlation was found between PWV and microvascular and macrovascular complications of T2DM. In summary, SGLT2 inhibitors are drugs with promising prospects for the improvement of vascular function and delaying vascular aging. Currently, the potential effects and mechanisms of SGLT2 inhibitors in this population are not completely understood and more studies are needed [83].

## 9. Osteoporosis and Fractures

The effect of SGLT2 inhibitors on bone metabolism, the development of osteoporosis, and the risk of fractures has been much debated. Until now, empaglifozin has not been shown to increase the risk of fractures [84]. Contradictory results have been found on dapaglifozin [85, 86]. An increased incidence of fractures was observed in patients treated with canaglizofin [87, 88, 89]. This effect could be explained by several mechanisms. Weight reduction may contribute to bone loss due to the direct effect of reduced soft tissue mass on bone [88]. In addition, the decrease in adipose tissue may reduce aromatase activity, decreasing estradiol levels and increasing bone turnover [90, 91, 92]. Finally, decreased sodium transport in the proximal convoluted tubule facilitates an increase in serum phosphorus levels, which would stimulate para thyroid hormone (PTH), enhancing bone turnover and increasing the risk of fractures [93].

Subsequently, a comprehensive assessment of the risks of amputation and fracture has been carried out, suggesting that the findings of the canaglifozin cardiovascular assessment study (CANVAS) program were chance observations rather than actual effects [94]. A recent meta-analysis shows that SGLT2 inhibitors do not modify the risk of fracture with statistically significant differences [95].

Currently, this association between SGLT2 inhibitors and changes in bone metabolism and fracture risk is considered inconclusive, although further studies are recommended. Importantly, T2DM itself adversely affects metabolism and bone strength and increases the risk of fracture in those affected [96].

## 10. Respiratory Diseases

Pulmonary disease is often present concomitantly in patients with T2DM, heart failure and/or chronic kidney disease. Evidence on the use of SGLT2 inhibitors in pulmonary pathology is sparse, but some currently existing data may provide guidance for future studies.

A meta-analysis of 9 RCTs concluded that the use of SGLT2 inhibitors can significantly reduce the occurrence of acute pulmonary edema (risk rate (RR) 0.51, 95% CI: 0.29 to 0.88), asthma (RR 0.57, 95% CI: 0.33 to 0.995), and sleep apnea syndrome (RR 0.35, 95% CI: 0.12 to 0.99); furthermore, their use may lead to trends towards reduced risk of chronic obstructive pulmonary ddisease (COPD) (RR 0.79, 95% CI: 0.61 to 1.02; *p* = 0.073) and pulmonary hypertension (RR 0.43, 95% CI 0.16 to 1.17; *p* = 0.098) [97].

In addition, another meta-analysis showed a significant association between the use of SGLT2 inhibitors and a reduction in the occurrence of infectious respiratory pathology (i.e., bronchitis, pneumonia, and respiratory tract infection). This could be related to the glucose-lowering efficacy of SGLT2 inhibitors. It also showed a significant association between the use of SGLT2 inhibitors and four types of non-infectious respiratory disorders (i.e., chronic obstructive pulmonary disease, non-small cell lung cancer, pleural effusion, and lung mass) [98].

Thanks to these findings, further research on SGLT2 inhibitors for the primary and secondary prevention of various respiratory disorders could be conducted.

## 11. Conclusions

SGLT2 inhibitors are drugs that have demonstrated efficacy in T2DM, heart failure, and chronic kidney disease. In the absence of further studies, our impression is that SGLT2 inhibitors are beneficial for the management of the main comorbidities that these patients often present. Some metabolic benefits of SGLT2 inhibitors are not limited to diabetics, but they also offer positive results in non-diabetic diseases, such as obesity, high blood pressure, or hyperuricemia. Other entities, such as hyperlipemia, PCOS or vascular aging need further studies to corroborate the efficacy of SGLT2 inhibitors in patients without diabetes mellitus. Randomized clinical trials are needed to better understand the benefit of SGLT2 inhibitors in the various comorbidities associated with T2DM, heart failure and chronic kidney disease.

## 12. Future Directions

Future research efforts are needed to understand the exact molecular mechanism responsible for the beneficial activity of SGLT2 inhibitors in different T2DM-associated comorbidities. This information could reveal the most suitable patients with T2DM to receive treatment with SGLT2 inhibitors.

All this could provide advantages as a result of a possible simplification of treatments that act in the management of different comorbidities and reduce the cardiovascular risk of this patient profile, regardless of the presence or absence of T2DM.
